# Factors Affecting Physical Activity of People with Knee Osteoarthritis in Southern Taiwan: A Multiple Logistic Regression Analysis

**DOI:** 10.1155/2022/4736231

**Published:** 2022-09-24

**Authors:** Min-Fen Hsu, Chun-Man Hsieh, Aih-Fung Chiu

**Affiliations:** ^1^Department of Nursing, Kaohsiung Veterans General Hospital Pingtung Branch, Pingtung, Taiwan; ^2^Department of Nursing, Meiho University, Pingtung, Taiwan; ^3^Department of Nursing, Tajen University, Pingtung, Taiwan

## Abstract

**Background:**

Physical activity (PA) is a basic and initiative conservative management for people with knee osteoarthritis (KOA). This study aimed to explore the potential indicators of PA levels in people with KOA.

**Methods:**

We designed a cross-sectional study where people with KOA were consecutively approached by the Orthopedic Outpatient Department in a hospital in southern Taiwan. People older than 50 years that could communicate and consent to the present study were enrolled. As a dependent variable, the Chinese version of the Physical Activity Scale for the Elderly (PASE-C) was used to assess the participant's PA levels. Considering differences in sex, a PASE-C score cut-off point of 140 for men and 120 for women was used. Participants were then divided into “active” and “inactive” groups. We measured independent variables consisting of the demographic and clinical characteristics, such as comorbidities measured by the Charlson Comorbidity Index (CCI), depression status measured by the Geriatric Depression Scale-5, body mass index, KOA history (<5, 5–<10, and ≥10 years), knee pain (unilateral or bilateral), the severity of symptoms measured by the Western Ontario and McMaster Universities Osteoarthritis Index, and 6-meter preferred walking speed. Multiple logistic regression was performed to identify significant relationships between PA among people with KOA.

**Results:**

We analyzed a total of 188 people with KOA (56 men and 132 women) with a mean age of 69.4 ± 7.9 (range: 51 to 90 years). Approximately 72.9% (*n* = 137) were categorized as “inactive PA,” while 27.1% (*n* = 51) of participants were categorized as “active PA” (male: 32.1%; female: 25.0%). Multiple logistic regression showed a positive association of 6-meter preferred walking speed with active PA (OR: 7.08; 95% CI:1.14–44.13), whereas advanced age and comorbidity (CCI≥1 vs. CCI<1) were negatively associated with active PA with an OR (95% CI) score of 0.91 (0.86–0.97) and 0.37 (0.15–0.87), respectively.

**Conclusions:**

People with KOA require appropriate lifestyle management to increase PA. Walking speed may be an effective factor for predicting PA among people with KOA. Healthcare providers treating KOA patients should be aware of their PA levels, especially those at risk.

## 1. Introduction

Knee osteoarthritis (KOA) is a very common, age-related degenerative joint disease characterized by the breakdown and loss of joint cartilage, subchondral bone degeneration, and synovitis. [1, 2] The estimated global prevalence of KOA is 16.0% (95% CI, 14.3%–17.8%) in individuals aged 15 and over and 22.9% (95% CI, 19.8%–26.1%) in individuals aged 40 and over. [3] People with KOA commonly suffer from pain, aching, stiffness, and associated mobility impairment or disability, leading to a poor quality of life. [4, 5] With an increasingly aging population, more attention to the issue of KOA is needed.

Physical activity (PA) is a basic initiative and conservative choice in promoting health and is considered a priority during KOA management[2, 6]. PA is defined as any bodily movement produced by skeletal muscles that require energy expenditure and can be occupational, athletic, or routine in nature. PA is considered an important, modifiable lifestyle behavior that can reduce pain and improve mobility in people with KOA[7–9]. Numerous arthritis guidelines and studies specifically report that people with KOA can benefit from improved symptoms and maintain health from moderate PA[10–12]. As the fourth leading cause of death worldwide, physical inactivity can increase the risk of noncommunicable diseases, such as cardiovascular or metabolic disorders[2, 13–15]. According to empirical studies, PA reportedly reduces pain and improves mobility in people with KOA[7–9]. People with higher PA showed higher functional performance than at baseline during a 1 or 2-year follow-up[16, 17]. In contrast, physical inactivity may increase morbidity and mortality[18]. However, most people with KOA do not meet recommended PA levels[19, 20]. These kinds of lifestyle modalities are typically underutilized in practice, despite being safe and essential. Considering the benefits of PA and the detrimental consequences of physical inactivity, paying more attention to the importance of PA is crucial.

Studies show that advanced age[21–23], female gender[23], depression[23, 24], increased BMI[23], increased comorbidities[23, 25], and symptom severity [23] negatively correlate with PA levels, while better social functioning (such as spousal support) [23], greater lower limb mobility, and faster gait speed [23, 26] can improve PA levels. From a public health perspective, assessing PA levels in people with KOA is essential for identifying contributing factors, thereby identifying people at risk of entering a sedentary lifestyle and proposing early intervention where necessary. To our knowledge, most published studies have been conducted in the Western world, and data from Asia are relatively limited. Therefore, the present study aimed to address the following research questions: “What PA levels are present in Taiwanese people with KOA, and what variables significantly correlate with active PA after adjusting for potential KOA-related variables?”

## 2. Methods

### 2.1. Study Design and Sample

We conducted a cross-sectional study with purposive sampling from July to August 2020. People with KOA who attended the orthopedic outpatient clinic of a hospital in southern Taiwan were recruited. Participation required people to (1) be clinically diagnosed with KOA without a replaced knee and (2) provide informed consent or, if unable to, proxy-informed consent from their substitute decision-maker. Patients were excluded if diagnosed with the inflammatory rheumatologic disease (e.g., rheumatoid arthritis), other severe diseases (e.g., severe cardiovascular diseases or cancer), or were unable to complete assessments due to other issues.

Using in-person interviews, all participants were asked to answer questions regarding their PA, demographic and clinical characteristics, such as the severity of KOA symptoms, and 6-meter preferred walking speed. The data collection process was noninvasive and performed by researchers and two trained research assistants. Ethical approval was obtained from the institutional review committee of the selected hospital (No. KSVGH20-CT6-09).

### 2.2. Outcome Measures

Self-reporting is the most commonly used method to measure PA in large observational studies. PA level, as the primary outcome of the present study, was assessed using the Chinese version of the Physical Activity Scale for the Elderly (PASE-C)[27], which was developed by Washburn et al. (1993) [28]. This scale contains 12 items assessing leisure (5 items: walking, light/moderate/strenuous sport, recreational activities, and muscle strength), household (6 items: light housework, heavy housework, home repairs, lawn work/yard care, outdoor gardening, and caring for another person), and occupational activities (1 item: work for pay or as a volunteer) for a 7-day period for older adults. Each activity was weighted and summed based on manual instructions, with higher scores indicating greater physical activity [27] because PA levels in our study sample were not normally distributed, a cut-off point for PASE scores of 140 for males and 120 for females was established, as suggested by Logan et al. (2013)[29]. Respondents below this threshold were classified as “inactive”, while the remaining participants were classified as “active”.

### 2.3. Demographic and Clinical Characteristics

We collected data regarding age, gender, an education level (year), marital status (living with or without a spouse), and clinical characteristics regarding comorbidities, depression, body mass index (BMI), self-reported KOA history (<5, 5–<10, and ≥10 years), site of knee pain (unilateral or bilateral), and severity of KOA symptoms. Comorbidities were measured using the Charlson Comorbidity Index (CCI) with a self-reported doctors' diagnosis method. [30] Depression was screened using the Chinese version of the Geriatric Depression Scale (GDS-5), with a score of 2 or more indicating a clinically positive diagnosis for depressive status [31]. BMI was calculated by measuring body weight and height. Participants with a BMI equal to or over 24.0 kg/m^2^ were considered obese, according to the WHO criteria. The severity of KOA symptoms was measured using the Western Ontario and McMaster Universities Osteoarthritis (WOMAC) Index. [32] The WOMAC Index is a widely used, proprietary set of standardized questionnaires, including pain (5 items), stiffness (2 items), and physical functioning of joints (17 items). The options for each response are none, mild, moderate, severe, or extreme, which are scored from 0 to 4, respectively. Greater total scores mean higher levels of perceived symptom severity, irrespective of present medication. In our study, the internal consistency reliability coefficient was 0.95.

### 2.4. Six-Meter Preferred Walking Speed

In our study, the 6-meter preferred walking speed was specifically chosen to assess PA levels. Participants were instructed to walk at their usual pace without running or jogging for a distance of 6 meters. Walking time and speed were calculated by dividing distance by time (in minutes).

### 2.5. Analysis

Descriptive statistics are reported as a number (percentage, %) for categorical variables and as mean ± standard deviation (SD) for continuous variables. When comparing inactive and active PA groups, Student's *t*-tests were used to compare mean values for continuous variables, and *χ*^2^ -tests were used to compare proportions of categorical variables. Any significant demographic and clinical characteristics and 6-m preferred walking speed according to bivariate analyses (Student's *t*-tests or *χ*^2^ tests) were used in multiple logistic regression using the enter method. This allowed us to adjust for known factors affecting active PA and identify significant independent factors among people with KOA. The results were reported as odds ratios (ORs) in 95% confidence intervals (CIs). For all statistical tests, SPSS for Windows 23.0 (IBM, Chicago, IL, US) was used and a *P* value of less than 0.05 was considered statistically significant.

## 3. Results

### 3.1. Participants

A total of 188 people with KOA (mean age 69.4 ± 7.9 years, range 51–90 years), consisting of 132 women and 56 men, were enrolled in this study. The mean PASE-C score was 93.0 ± 65.1 (range: 0–398.5). We used a score cut-off point of 140 for men and 120 for women [29] to distinguish sex differences when measuring PA levels. A total of 137 (72.9%) were categorized as “inactive PA”, while 51 (27.1%) participants were categorized as “active PA” (male: 32.1%; female: 25.0%).

Table 1 shows that participants with inactive PA were older, lived without a spouse, showed higher comorbidities (CCI≥1), had longer self-reported KOA histories and showed apparent bilateral knee pain compared with the active PA group (*P* < 0.05). No significant differences between groups were found for the other variables.  Table 2 shows that participants in the inactive PA group were older, had higher WOMAC index scores (more severe KOA symptoms), and preferred slower walking speeds than in the active PA group (*P* < 0.05).

All variables with significant differences between groups in Tables 1 and 2 were included in the multiple logistic regression analysis (enter method).  Table 3 shows that the 6-meter preferred walking speed was a positive association with active PA (OR: 7.08; 95% CI:1.14–44.13), whereas advanced age and comorbidity (CCI≥1 vs. CCI<1) showed a negative relationship with active PA, with an OR (95% CI) of 0.91 (0.86–0.97) and 0.37 (0.15–0.87), respectively. There were no significant differences in marital status (living with/without a spouse), OA history (<5 years, 5–<10 years, and ≥10 years), pain site (unilateral or bilateral), and symptom severity are assessed using the WOMAC index. Using the Hosmer–Lemeshow goodness-of-fit test (*χ*^2^ = 9.39, df = 8, *P* = 0.311), which did not reach significance, the regression model showed ideal goodness of fit. Cox and Snell and Nagelkerke *R*^2^ values were 0.21 and 0.30, implying that approximately 21.0%–30.0% of the variance in PA was accounted for by this model. Tests on the Wald values for age, CCI score, and 6-meter preferred walking speed resulted in *P* values ≤ 0.05, indicating that these three variables are independent factors.

## 4. Discussion

Our study assessed PA levels using the PASE-C questionnaire. The mean PASE-C (SD) score was 93.0 (±65.1), with a range of 0 to 398.5. A previous study using the same PASE-C questionnaire found a mean ± SD of 104.4 ± SD 47.1 in a community-dwelling of older people. [33] This result was higher than our study sample, indicating with a certain degree of confidence that our samples had relatively lower PA levels. In contrast, lower PASE-C scores were reported in people living in community and assisted living facilities [34], or in outpatients diagnosed with chronic obstructive pulmonary disease and aged 60 years or more[35]. Although lower PA levels are seen in frail people or those with other chronic diseases, the inactive lifestyle in people with KOA is still apparent. Considering the benefits of KOA patients engaging in PA compared with a sedentary lifestyle, this is likely to contribute to negative health consequences[18]. Healthcare providers should appropriately monitor, encourage, and promote an increase in PA levels in KOA patients. Efforts to develop interventions and strategies for engaging in active PA should also be created.

After controlling all variables, we found that faster walking speed still showed a strong relationship with active PA. Walking speed has been termed “the sixth vital sign” [36] due to its wide predictive capacity in numerous health issues, such as disability and frailty [37, 38]. In our study, 6-meter preferred walking speed was significantly associated with active PA, which is congruent with a previous study of 67 older adults in a community, where walking speed effectively predicted daily ambulatory activity (steps per day) in community-dwelling older adults and accounted for 51% of explained variation[26]. Our study further stressed the relevance of walking speed in PA assessments in people with KOA. Indeed, as a basic daily activity and form of exercise for most people, walking can explain or predict PA levels and reduce KOA symptoms. However, as a PA indicator, walking speed was relatively limited. Given the ease of observing walking speed in clinical settings or communities, our findings imply that healthcare providers should pay more attention to KOA people with slower walking speeds. Further work clarifying this cause-effect relationship may be required.

As expected, advanced age was negatively associated with active PA (*P*=0.002). Each added year of life was associated with a 9% decrease in OR of PA in this study sample. This outcome was congruent with previous studies[21–23], in which older adults showed a more sedentary lifestyle. Age-related changes in muscle architecture and metabolism [39] may lead to physical inactivity, which is a concern with the aging society and the burden of KOA in Taiwan. Further studies are needed to develop an effective PA program to improve health and avoid adverse outcomes for older people, especially those with KOA.

People with osteoarthritis (OA) commonly show various comorbidities that are likely to influence PA behavior[25]. In the present study, people with KOA with a CCI≥1 showed reduced OR to have active PA compared with those with CCI<1. Although we focused on people with KOA and used the CCI scale to assess comorbidity, our findings support the results of previous studies in which comorbidity presence was associated with an incrementally lower PA in people with knee, hip, or other kinds of OA, especially in those with respiratory or cardiovascular comorbidities[23, 25]. Based on an analysis of individual diseases, we found that 16% (*n* = 30) had cardiovascular disease and 19.1% (*n* = 36) had diabetes, both of which showed significant differences between active and inactive groups according to bivariate analyses(Table 1). This outcome implies that activity intolerance caused by cardiovascular or other diseases may complicate or restrict daily PA in people with KOA. In clinical practice, a greater focus on complicated issues caused by the combination of KOA and cardiovascular or other diseases may be necessary.

For variables relating to KOA, such as self-reported KOA history, pain site (unilateral or bilateral) or the WOMAC score, only functioned as significant factors in the bivariate analyses but not in the multiple logistic regression analyses. Empirical studies have demonstrated that increasing PA or exercise can reduce pain and improve mobility in people with KOA[7–9]. However, debilitating pain symptoms caused by KOA often limit walking ability[5], which can translate to inactivity[40]. Many people worry that high PA levels may cause osteoarthritis, aggravate KOA symptoms, or inflict further harm to injured joints [41], resulting in a sedentary lifestyle or general reluctance to exercise. Knee pain is indeed associated with moderate intensity activity but only corresponds to overloaded [42]. Increased PA may be beneficial by preventing cartilage degeneration, inhibiting inflammation, and preventing loss of subchondral bone, and metaphyseal bone trabeculae[43]. Therefore, considering the benefits of PA and damage to joints caused by higher-intensity exercise, activity recommendations for KOA patients need to be adjusted based on disease severity to improve suitability for people with KOA and avoid joint damage caused by excessive exercise[44].

In previous studies, spousal support for PA was a key factor in initiating and maintaining PA in the general population[45,46]. Spousal support was identified as a possible factor influencing PA[23]. In the present study, the bivariate analysis indicated that participants who lived with spouses had a higher proportion of active PA, but this effect disappeared after controlling for other variables. However, the influence of social and family support should not be ignored and is worth further exploration.

Our study sheds light on factors affecting PA in people with KOA, however, it was subject to some limitations. First, there exists no comparable standard to use as supporting evidence for the validity of PASE-C score outcomes in older adults. This self-reported questionnaire may also be prone to recall bias, misclassification, and over- or underestimation of PA levels. Further research using objective PA measures, such as accelerometers cached in wearable devices, should allow for more accurate outcomes. Second, since the cross-sectional nature of our study prevents inferring causation from our results, any relationships should be carefully interpreted before implying causation. Further longitudinal studies may be required to explore these relationships. Third, our sample size was relatively small at 188 KOA patients, all of whom were enrolled from the OPD of the same hospital. Caution is therefore needed when generalizing our results to other populations or patients in different hospitals. Finally, the fact that only 21–30% of the variance can be explained by the multiple logistic regression model, implies that there are some variables that can potentially explain the remaining variance. Further studies regarding advancing possible variables might be needed. Despite these limitations, our study provides useful information for policymakers, health planners, and health care providers by highlighting potential factors influencing PA levels among people with KOA. By revealing these significant relationships, this study advances our present understanding of PA status among people with KOA.

## 5. Conclusion

Increasing PA is an important goal for people with KOA, but patients included in this study showed low PA levels. Our findings suggest that interventions aimed at increasing PA in people with KOA are essential, especially for people of advanced age or with comorbidities or lower preferred 6-meter walking speed.

## 6. Disclosure

The funding organization had no role in the conduct of the study, collection, interpretation of data, or decision to submit the manuscript.

## Figures and Tables

**Table 1:** The comparison of categorical variables between inactive and active physical activity groups in people with KOA (*n* = 188).

Variables	Total *n* = 188		Inactive PA *n* = 137		Active PA *n* = 51		*P* value
Sex							0.314
Men	56	(29.8)	38	(27.7)	18	(35.3)	
Women	132	(70.2)	99	(72.3)	33	(64.7)	
Age range							0.001^*∗*^
50 to < 65 years	56	(29.8)	32	(23.4)	24	(47.1)	
65 to < 75 years	82	(43.6)	60	(43.8)	22	(43.1)	
≥75 years	50	(26.6)	45	(32.8)	5	(9.8)	
Marital status							0.040^*∗*^
Without spouse	78	(41.5)	63	(46.0)	15	(29.4)	
With spouse	110	(58.5)	74	(54.0)	39	(70.6)	
Comorbidities							0.014^*∗*^
CCI <1	117	(62.2)	78	(56.9)	39	(76.5)	
CCI ≥1	71	(37.8)	59	(43.1)	12	(23.5)	
Types of comorbidities							
Hypertension							
No	103	(54.8)	70	(51.1)	33	(64.7)	0.095
Yes	85	(45.2)	67	(48.9)	18	(35.3)	
Cardiovascular							
No	158	(84.0)	110	(80.3)	48	(94.1)	0.021^*∗*^
Yes	30	(16.0)	27	(19.7)	3	(5.9)	
Diabetes							
No	152	(80.9)	106	(77.4)	46	(90.2)	0.047^*∗*^
Yes	36	(19.1)	31	(22.6)	5	(9.8)	
Depression status							0.064
GDS-5 <2	158	(84.0)	111	(81.0)	47	(92.2)	
GDS-5 ≥2	30	(16.0)	26	(19.0)	4	(7.8)	
Body mass index							0.669
<24 kg/m^2^	56	(29.8)	42	(30.7)	14	(27.5)	
≥24 kg/m^2^	132	(70.2)	95	(69.3)	37	(72.5)	
Self-reported arthritis history							0.008^*∗*^
<5 years	91	(48.4)	57	(41.6)	34	(66.7)	
5 to 10 years	56	(29.8)	45	(32.8)	11	(21.6)	
≥10 years	41	(21.8)	35	(25.5)	6	(11.8)	
Site of pain knee							0.038^*∗*^
Unilateral pain	91	(48.4)	60	(43.8)	31	(60.8)	
Bilateral pain	97	(51.6)	77	(56.2)	20	(39.2)	

^*∗*^ *P* < 0.05. *χ*2 -tests were used to compare proportions of categorical variables. SD, standard deviation; KOA, knee osteoarthritis; CCI, Charlson Comorbidity Index; GDS, Geriatric Depression Scale-5.

**Table 2:** The comparison of continuous variables between inactive and active physical activity groups in people with KOA (*n* = 188).

Variables	Total *n* = 188		Physical activity				
			Inactive *n* = 137		Active *n* = 51		*P* alue
Age (years)	69.37	±7.92	70.97	±7.83	65.06	±6.46	<0.001^*∗*^
WOMAC score	43.86	±14.57	45.49	±15.30	39.47	±11.38	0.011^*∗*^
6-m preferred walking speed (m/sec)	0.97	±0.26	0.92	±0.27	1.09	±0.18	<0.001^*∗*^

Data are mean ± standard deviation. Student's *t*-tests were used to compare the mean values. ^*∗*^*P* < 0.05. WOMAC index: McMaster Universities Osteoarthritis Index.

**Table 3:** Results of multiple logistic regressions analyses of active physical activities among people with KOA (*n* = 188).

Variables	B	S,E.	Wald value	OR	(95% CI)	*P* value
Age^†^ (year)	−0.093	0.031	9.248	0.91	(0.86–0.97)	0.002^*∗*^
Marital status						
Without spouse (*n* = 78)				1		
With spouse (*n* = 110)	0.129	0.410	0.098	1.14	(0.51–2.54)	0.754
Comorbidities						
CCI <1 (*n* = 117)				1.00		
CCI ≥1 (*n* = 71)	−1.006	0.441	5.202	0.37	(0.15–0.87)	0.023^*∗*^
Self-reported history of OA						
<5 years (*n* = 91)				1		
5 to 10 years (*n* = 56)	−0.455	0.451	1.017	0.64	(0.26–1.54)	0.313
≥10 years (*n* = 41)	−0.542	0.556	0.952	0.58	(0.20–1.73)	0.329
Site of pain knee						
Unilateral (*n* = 91)				1.00		
Bilateral (*n* = 97)	−0.594	0.390	2.317	0.55	(0.26–1.19)	0.128
WOMAC index score^†^	−0.013	0.017	0.548	0.99	(0.95–1.02)	0.459
6 m preferred walking speed (m/sec)^†^	1.958	0.933	4.399	7.08	(1.14–44.13)	0.036^*∗*^
Constant	4.636	2.814	2.714	103.13		0.099

^*∗*^ *P* < 0.05;^†^ indicates continuous variable. CCI, Charlson Comorbidity Index; goodness of fit: *χ*2 = 44.24,*P* < 0.001; Hosmer–Lemeshow *χ*^2^ = 9.39 df = 8, *P*=0.311. Strength of association: Cox and Snell *R*^2^ = 0.21; Nagelkerke *R*^2^ = 0.30.

## Data Availability

The data used to support the findings of this study are included within the article.
